# Evaluation of Microbial Load in Oropharyngeal Mucosa from Tannery Workers

**DOI:** 10.1016/j.shaw.2014.09.003

**Published:** 2014-10-07

**Authors:** Diana C. Castellanos-Arévalo, Andrea P. Castellanos-Arévalo, David A. Camarena-Pozos, Juan G. Colli-Mull, María Maldonado-Vega

**Affiliations:** 1 Departamento de Investigación en Ambiental, Centro de Innovación Aplicada en Tecnologías Competitivas (CIATEC, AC), León, Guanajuato, Mexico; 2 Departamento de Biología, Instituto Tecnológico Superior de Irapuato (ITESI), Irapuato, Guanajuato, Mexico; 3 Dirección de Enseñanza e Investigación, Hospital Regional de Alta Especialidad del Bajío. León, Guanajuato, México

**Keywords:** diarrheal diseases, opportunistic microorganisms, oropharyngeal mucosa, respiratory diseases, tannery's working environment

## Abstract

**Background:**

Animal skin provides an ideal medium for the propagation of microorganisms and it is used like raw material in the tannery and footware industry. The aim of this study was to evaluate and identify the microbial load in oropharyngeal mucosa of tannery employees.

**Methods:**

The health risk was estimated based on the identification of microorganisms found in the oropharyngeal mucosa samples. The study was conducted in a tanners group and a control group. Samples were taken from oropharyngeal mucosa and inoculated on plates with selective medium. In the samples, bacteria were identified by 16S ribosomal DNA analysis and the yeasts through a presumptive method. In addition, the sensitivity of these microorganisms to antibiotics/antifungals was evaluated.

**Results:**

The identified bacteria belonged to the families Enterobacteriaceae, Pseudomonadaceae, Neisseriaceae, Alcaligenaceae, Moraxellaceae, and Xanthomonadaceae, of which some species are considered as pathogenic or opportunistic microorganisms; these bacteria were not present in the control group. Forty-two percent of bacteria identified in the tanners group are correlated with respiratory diseases. Yeasts were also identified, including the following species: *Candida glabrata*, *Candida tropicalis*, *Candida albicans*, and *Candida krusei*. Regarding the sensitivity test of bacteria identified in the tanners group, 90% showed sensitivity to piperacillin/tazobactam, 87% showed sensitivity to ticarcillin/clavulanic acid, 74% showed sensitivity to ampicillin/sulbactam, and 58% showed sensitivity to amoxicillin/clavulanic acid.

**Conclusion:**

Several of the bacteria and yeast identified in the oropharyngeal mucosa of tanners have been correlated with infections in humans and have already been reported as airborne microorganisms in this working environment, representing a health risk for workers.

## 1 Introduction

Mexico is ranked among the 10 largest leather manufacturers in the world; the Mexican state of Guanajuato is responsible for 65% of the tanning and finishing of leather products. The tannery industry is one of the largest economic activities in the city of León, Guanajuato. Approximately 700 leather tanneries are located in this city, which range significantly in sophistication; some consisting of small family-run businesses, with < 10 employees and having minimal infrastructure, to modern manufacturing facilities with > 100 employees. The health condition of tannery employees is adversely affected by the working environment, which includes physical, chemical, and biological factors, and dust and fumes present in the atmosphere [1]. The severity of the effect on health varies on individual physical characteristics including allergen sensitivity, immune capacity, and exposure to the contaminants (frequency, duration, and type).

The unprocessed animal hides, the raw material used in the tannery industry that has a high moisture content as well as growth facilitating nutrients (carbohydrates, fats, and proteins), provide an ideal medium for the rapid reproduction of microorganisms [2]. Prior research shows a significant correlation between microbial concentration and temperature and relative humidity [3], because most of the bacteria and fungi require specific environmental conditions to proliferate. The processing of the animal hides requires significant water usage; causing a high-humidity working environment. When combined with low-oxygen concentration, elevated temperatures, and low air circulation, the high-humidity environment acts as a catalyst for microorganism propagation [4].

The most frequent bacteria in unprocessed hides include: *Escherichia coli*, *Staphylococcus epidermidis*, *Morganella morganii*, *Proteus mirabilis*, *Proteus vulgaris*, *Bacillus anthracis*, *Bacillus subtilis*, and *Bacillus mycoides* [2]. Tetanus, anthrax, leptospirosis, epizootic aphtha, Q fever, and brucellosis are examples of diseases that workers have contracted during the tanning process because of contaminated hides [5].

In addition to bacteria, filamentous fungi were also identified, belonging to the species: *Penicillium commune*, *Penicillium glaucum*, *Penicillium wortmannii*, *Penicillium frequentans*, *Aspergillus niger*, *Aspergillus flavus*, *Aspergillus oryzae*, and *Aspergillus fumigatus.* Other genera found include: *Alternaria*, *Cladosporium*, *Trichoderma*, *Fusarium*, *Aureobasidium*, and *Scopulariopsis* [2].

The possibility of contracting an infection is a *Aspergillus fumigatus* constant hazard for the tannery employees, because the hide serves as a medium for numerous microorganisms; several of these organisms also have been identified as airborne microorganisms [6,7], related to the development of respiratory disease contracted by inhalation [8]. Several antibiotic resistant bacteria have been strongly correlated with respiratory and diarrhea illnesses. In addition, yeasts belonging to the genus *Candida* were also identified. Normally found in the oral cavity, these bacteria have also been correlated with autoimmune diseases and immunosuppressed patients [9]. Respiratory diseases caused by inhalation of mold spores include atopic asthma, rhinitis, hypersensitivity pneumonitis, allergic bronchopulmonary aspergillosis, allergic fungal sinusitis, and other detrimental health effects like infections and allergic reactions are well documented [8,10,11].

During the period from 1998 to 2001, a study conducted by the Guanajuato Secretary of Health identified 58 locations in the city of León, with increased mortality risk due to diarrhea and respiratory diseases. These locations were included in industrial zones and areas with local water sources heavily contaminated with tannery effluent. In addition, the Guanajuato Secretary of Health also reported 146,930 cases of respiratory infections and 27,530 cases of acute diarrhea, which constituted the two predominant symptoms of disease in the State during 2012 [12].

Our department carried out previous studies in 23 tanneries and a control site in the city of León, Mexico [13]. In this study the microbiological quality in the indoor air was evaluated following the methodology described by the National Institute of Safety and Hygiene at Work NTP-409 and NTP-299 of the Ministry of Labor and Social Affairs of Spain [14,15]. The average fungi concentration by tannery ranged from 100 colony-forming units (CFU)/m^3^ to 10,000 CFU/m^3^, and in the case of bacteria, the average load ranged from 400 CFU/m^3^ to 6,000 CFU/m^3^. The fungi and bacterial loads found in the control site were significantly lower, at ≤ 300 CFU/m^3^ and ≤ 120 CFU/m^3^, respectively. Because of the lack of Mexican guidelines that limit values of airborne microorganisms for indoor environments, these results were compared with particular European countries' guidelines, specifically the Swedish requirements [16]. The results showed that 87% of the studied tanneries had a bacteria load < 500 CFU/m^3^ and 83% of these had a fungi load < 300 CFU/m^3^. The bacteria and fungi loads in the control site were lower than the Swedish limits. The indoor/outdoor ratio (I/O), an indicator of air quality [17] revealed that 43.5% of the studied tanneries had a poor air quality indoors due to bacterial load, similarly 52.2% of the studied tanneries, had a poor air quality indoors due to fungal load. Additionally, this study revealed the presence of airborne pathogens hazardous to humans in the indoor tannery environment.

The objective of the current study was to quantify and identify the microorganisms present in the oropharyngeal mucosa of a group of tannery employees versus those present in the oropharyngeal mucosa of a control group, in order to investigate a possible correlation between the work environment and the health status of workers.

## 2 Materials and methods

### 2.1 Sampling

This study took place at the tannery industrial zone in León, México. The study was developed during September 2012 to May 2013. The selected tanneries were operational during the study period. One study group of tannery workers (tanners) and one control group of automotive industry workers were analyzed. Oropharyngeal samples from the tanners group (*n* = 19) were taken during October–November 2012. Samples from the control group were taken during December–February 2012. The samples were taken in a clinical environment, in the Laboratory of Public Health, Guanajuato State.

Oropharyngeal mucosa samples were taken through a sterile hyssop, using a Stuart medium and preserved in cold for transport to the laboratory.

### 2.2 Processing of samples

The samples were then cultured on solid medium in Petri dishes, for which each hyssop corresponding to each worker was streaked on solid medium by massive striae technique. The solid media used were Brilliant Green Bile Agar (BGBA, Becton, Dickinson and Company), Tryptic Soy Agar (TSA, Becton, Dickinson and Company), and Sabouraud Dextrose Agar (SDA, Becton, Dickinson and Company) (BD, Becton, Dickinson and Company), Cuautitlán Izcalli, Estado de México, México, to enterobacteria, bacteria, and yeast growth, respectively. Enterobacteria and bacteria were then incubated at 37°C for 48 hours, and yeast were incubated at 25°C for 72 hours. At the conclusion of the incubation period, a counting was conducted and reported as CFU/mL. Each bacterium and yeast was macroscopically differentiated, based on the following parameters: whole shape, size, edge/margin, color, opacity, elevation, surface, and consistency.

### 2.3 Differentiation of microorganisms

Each bacterium and yeast macroscopically differentiated was isolated and identified; in this process each bacterium was streaked on a plate containing TSA and the yeasts were streaked on SDA plates. Bacteria were incubated at 37°C for 48 hours, and yeast at 25°C for 72 hours. Then, each isolated bacterium was inoculated in 5 mL of Tryptic Soy Broth (TSB, Becton, Dickinson and Company) and incubated at 37°C through shaking (250 rpm) all night. Each yeast was inoculated in 5 mL of Sabouraud Dextrose Broth (SDB- Becton Dickinson and Company) and incubated at 25°C through shaking (250 rpm) during a 48–72-hour period.

At the end of the incubation period, the identification of bacteria was performed by DNA extraction, which was carried out using the kit ZR Fungal/Bacterial DNA Kit (Zymo Research, Irvine, USA). Amplification of the 16S ribosomal DNA (rDNA) gene was conducted using the primers: Fd1: 5′- CCG AAT TCG ACA GAG TTT GAT CCT GGC TCA G 3′ y Rd1: 5′ CCC GGG ATC CAA GCT TAA GGA GGT GAT CCA GCC 3′ (Invitrogen products by Accesorios para Laboratorios, S.A. de C.V. Cuajimalpa, Santa Fe, D.F., México) to amplify a segment of approximately 1,500 pb [18]. The polymerase chain reaction (PCR) was scheduled by the following process: 94°C for 5 minutes; and 40 cycles of 94°C for 50 seconds, 55°C for 1 minute and 72°C for 2 minutes; and a final extension of 10 minutes at 72°C. To confirm the extraction and amplification, a 1% agarose gel electrophoresis was conducted. The amplified purification was performed using kit UltraClean PCR Clean-Up, Mo Bio Laboratories By Científica Senna, SA de CV Cuauhtemoc, D.F., México.

The identification of yeast was performed by DNA extraction, which was carried out using the kit ZR Fungal/Bacterial DNA Kit (Zymo Research). The 5.8S rDNA gene was amplified under the following conditions: 96°C for 5 minutes; 40 cycles of 94°C for 30 seconds, 58°C for 30 seconds, and 72°C for 30 seconds; and a final duration of 5 minutes at 72°C, using primers ITS1: 5′ -TCC GTA GGT GAA CCC TGC GG and ITS2: 5′ -GCT GCG TTC TTC ATC GAT GC that amplified a segment of 250 bp of the gene 5.8S rDNA [19]. The results of extraction and PCR were confirmed doing an agarose gel electrophoresis of 1%. The amplified genes where then purified using the kit UltraClean PCR Clean-Up, Mo Bio Laboratories By Científica Senna, SA de CV Cuauhtemoc, D.F., México.

Additionally, a presumptive identification of yeast was performed by CHROMagar Candida, catalog 254093 Mexico, BD Becton Dickinson By Industrial Kem de León SA de CV León, Guanajuato, México. For this process, each of the yeasts were streaked on ChromAgar and then incubated at 37°C for 48 hours. The Petri dishes were then observed to identify the developed color for each individual yeast. *Candida albicans* developed a green color, *Candida tropicalis* developed a blue color, *Candida krusei* developed a pink color, other *Candida* species developed an ivory–violet color, and other genera such as: *Trichosporon* species developed a light blue (Cerebriforme and aerial mycelium), *Saccharomyces* species developed a creamy violet color, and *Cryptococcus* developed a beige color [20]. *Candida albicans* ATCC 90028 was used as a positive control (DIBICO-Mexico, DIBICO SA de CV, DF, México).

### 2.4 Sequencing of amplified genes

Sequencing was performed by the National Laboratory of Genomics for Biodiversity of CINVESTAV, Mexico, Irapuato, Guanajuato, México. Molecular identification was made by comparing the sequences obtained against all the nucleotide sequences reported in the National Center for Biotechnology Information, NCBI (http://www.ncbi.nlm.nih.gov).

### 2.5 Determination of antimicrobial susceptibility

Bacterial sensitivity was determined by disk diffusion method, against different antibiotics, including: piperacillin/tazobactam (TZP); interpretation data are shown in the Table 1. *Escherichia coli* ATCC 35218 was used as the quality control.

Each isolated enterobacterium was resuspended in buffer saline and its turbidity adjusted to 0.5 McFarland scale. Each resuspended enterobacteria was streaked on Mueller-Hinton agar with a sterile hyssop by massive striae. Diffusion disks containing the antibiotics were placed on the agar surface and incubated at 35°C over 18 hours [21]. The Mueller-Hinton culture media was prepared as indicated by the manufacturer's directions (BD Mexico: Becton, Dickinson and Company).

Yeast sensitivity to fluconazole was determined by a disk diffusion method, using *C. albicans* ATCC 90028 as quality control. The interpretation data are shown in Table 2.

Each yeast was resuspended in buffer saline and its turbidity adjusted to 0.5 McFarland scale, and each was streaked on Mueller-Hinton agar with a sterile hyssop by massive striae. Diffusion disks containing the fluconazole were placed on the agar surface and incubated at 35°C for 24 hours [22]. The Mueller-Hinton BD media was supplemented with glucose-methylene blue at a final concentration of 40% glucose and 10 μg/mL of methylene.

## 3 Results

### 3.1 Sampling

A total of 19 tannery workers and 20 workers of a control group agreed to participate in the study. Thus, 39 oropharyngeal mucosa samples were analyzed in the Microbiology Laboratory of CIATEC AC (Centro de Innovación Aplicada en Tecnologías Competitivas, AC) León, Guanajuato, México. All study participants were male. A result report was provided to each worker participant at the conclusion of the study.

### 3.2 Processing of samples

**Fig. 1:**
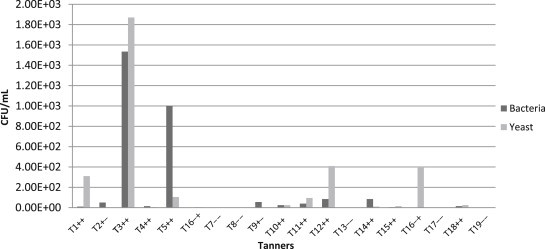
Bacteria and yeasts in bucopharinx mucosa samples from tanners. CFU/mL, colony-forming unit per milliliter; T1–T19, participating workers; ++, positive bacteria growth/positive yeast growth; +−, positive bacteria growth/negative yeast growth; −+, negative bacteria growth/positive yeast growth; −−, negative bacteria growth/negative yeast growth.

**Fig. 2:**
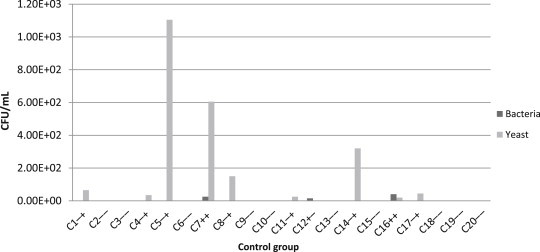
Bacteria and yeasts in bucopharinx mucosa samples from workers of the control group. CFU/mL, colony-forming unit per milliliter; C1–C20, participating workers; ++, positive bacteria growth/positive yeast growth; +−, positive bacteria growth/negative yeast growth; −+, negative bacteria growth/positive yeast growth; −−, negative bacteria growth/negative yeast growth.

The average bacterial load from oropharyngeal mucosa in the tanners group was 2.43 × 10^2^ CFU/mL and the average fungal load was 2.73 × 10^2^ CFU/mL, reaching values of up to 1.5 × 10^3^ CFU/mL and 1.8 × 10^3^ CFU/mL for bacteria and yeast, respectively (Fig. 1). The results of the control group showed an average bacterial load of 2.73 × 10^1^ CFU/mL and an average fungal load of 2.63 × 10^2^ CFU/mL, reaching values of up to 4.0 × 10^1^ CFU/mL and 1.1 × 10^3^ CFU/mL for bacteria and yeast, respectively (Fig. 2).

Additionally, in the tanners group (*n* = 19), 63% had a positive result to bacterial growth and 63% had a positive result to fungal growth (Table 3). In the control group (*n* = 20), 15% and 45% of employees had a positive result to bacterial and fungal growth, respectively.

**Fig. 3:**
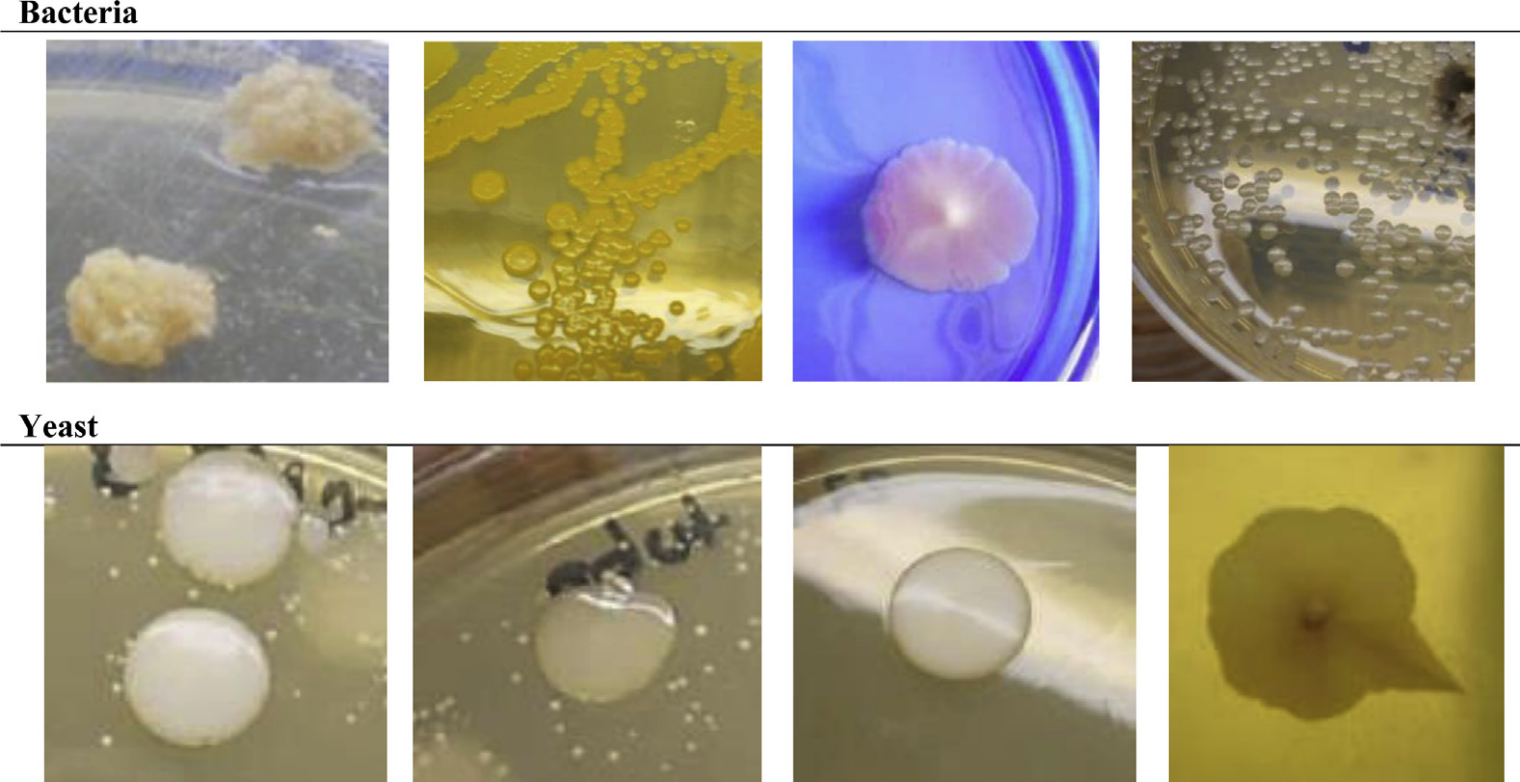
Based on macroscopic differentiation, bacteria and yeast are shown, in which can be seen different shapes, sizes, edges, colors, elevation, and surfaces.

**Fig. 4:**
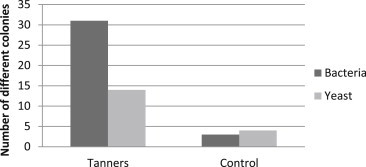
Macroscopic diversity of bacteria and yeast in oropharyngeal mucosa from tanners versus control group.

With respect to macroscopic differentiation, oropharyngeal samples from tanners revealed 31 different colonies of bacteria, and 14 different colonies of yeast (some bacteria and yeast are shown in Fig. 3), whereas the control group revealed three different colonies of bacteria and four different colonies of yeast (Fig. 4).

### 3.3 Identification of microorganisms

**Fig. 5:**
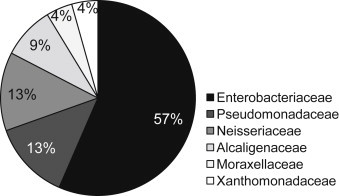
Bacterial families identified in oropharyngeal mucosa from the tanners group.

Only 74% of bacteria could be identified, of which the families detected included: Enterobacteriaceae, Pseudomonadaceae, Neisseriaceae, Alcaligenaceae, Moraxellaceae, and Xanthomonadaceae; of these, Enterobacteriaceae was the most abundant (57%; Fig. 5).

Identified species belonging to the Enterobacteriaceae family included: *Klebsiella pneumoniae, Enterobacter aerogenes, Enterobacter cloacae, Proteus vulgaris, Rahnella aquatilis, Citrobacter murliniae, Kluyvera ascorbata, Enterobacter asburiae, E. coli, and Serratia marcescens*.

The remaining bacteria found in the tanners group belong to other families, including: Pseudomonadaceae (*Pseudomonas brenneri*, *Pseudomonas psychrotolerans*, and *Pseudomonas aeruginosa*); Neisseriaceae (*Neisseria bacilliformis* and *Neisseria subflava*); Alcaligenaceae (*Achromobacter xylosoxidans* and *Alcaligenes faecalis*); Moraxellaceae (*Acinetobacter johnsonii*); and Xanthomonadaceae (*Stenotrophomonas maltophilia*). The bacteria identified in the control group included *Neisseria polyccharea* strain and *Acinetobacter calcoaceticus* (Table 4).

The presumptively identified yeasts by CHROMagar included: *Candida glabrata* (57%), *C. albicans* (21%), *C. tropicalis* (7%), and *C. krusei* (7%); 7% did not grow. In the control group, the identified species included *C. glabrata* (75%) and *C. albicans* (25%). *C. albicans* ATCC 90028 was used as quality control. The identified yeast by sequencing of 5.8S rDNA gene was *C. albicans* (Table 5).

### 3.4 Determination of antimicrobial susceptibility

**Fig. 6:**
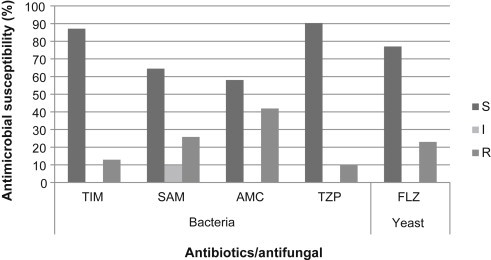
Antimicrobial susceptibility test of microorganisms identified in tanners. AMC, amoxicillin/clavulanic acid; FLZ, fluconazole; I, intermediate; R, resistant; S, Sensitive; SAM, ampicillin/sulbactam; TIM, ticarcillin/clavulanic acid; TZP, piperacillin/tazobactam.

The isolated bacteria from samples of oropharyngeal mucosa of tannery workers showed a varying response (percentage) of resistance to the antibiotics tested (Fig. 6). Ten percent of the bacteria were resistant to TZP, 13% to ticarcillin/clavulanate (TIM), 26% to ampicillin/sulbactam (SAM), and 42% to amoxicillin/clavulanic acid (AMC).

Bacteria susceptible to the four antibiotics tested included *Kluyvera ascorbata*, *R. aquatilis*, *Citrobacter murliniae*, *K. pneumonia*, *Stenotrophomonas maltophilia*, *P. vulgaris*, *A. faecalis*, *A. johnsonii*, *N. bacilliformis*, *N. subflava*, *P. psychrotolerans*, *A. xylosoxidans*, and *E. coli*. Other bacteria showed a variable response to the tested antibiotics (Table 6).

Eight bacteria were not identified, of which four showed susceptibility to all antibiotics assayed, three were resistant to all antibiotics assayed, and one was resistant to SAM only (data not shown).

Therefore, TZP presented a better antimicrobial response with 90% effectiveness, followed by TIM (87%), SAM (65%), and AMC (58%). In the control group, three bacteria were isolated. Two were resistant to the four antibiotics evaluated. These results are significant, demonstrating the effectiveness of the antibiotics evaluated.

With respect to the yeast susceptibility, 13 yeasts were isolated from tannery workers; of these, 23% showed resistance to the antifungal fluconazole (Table 7). In the control group, four yeasts were isolated of which 50% were resistant.

## 4 Discussion

The presence of microorganisms in the human body is normal; in fact, many microorganisms have been reported naturally inhabiting in the oropharyngeal mucosa [23]. In this study, both groups (tanners and control workers) had a microbiological load; however, in the case of bacterial load, the difference between the two groups was one order of magnitude. The difference becomes more significant if the higher bacterial loads are compared; this was 10^3^ in tanners and 10^1^ in control workers. In addition, both groups differ with respect to the fungal load; 63% of tanners had growth of fungi whereas 45% of workers in the control group had growth.

This difference of microbial load in tanners and control workers is partly caused by the working environment, to which tanners are exposed for long time periods (typically 8–12 h/d). The working environments (tannery and automotive) are significantly different, primarily because of the raw material (the animal hides) used in the tannery industry, which serves as a transport mechanism and food source for many microorganisms [2]. In addition, other factors are the indoor environmental conditions of each work site. Tanneries are characterized by an environment with high relative humidity and low air flow, which favors the propagation of microorganisms [4], leading to high microbial loads in indoor manufacturing environments.

Based on the results of our previous study [13], the relative humidity registered in the tannery environment reached up to 80%, whereas the relative humidity registered in the work site of control workers reached 32%. This is because of high water consumption during the tanning process, which promotes a damp environment. In addition; the wind speed (ventilation) was measured. Generally, this parameter was close to 0.0 m/s; however, the highest value registered in tanneries was 0.7 m/s and in the work site of the control group was 2.2 m/s. Therefore, both working environments are very different and can be correlated with the airborne microbiological load and thus, with the microbiological load found in the oropharyngeal mucosa from workers.

Also, fungal and bacterial load in the indoor environment was reported. Fungal load in indoor tanneries reached values up to 1 × 10^4^ CFU/m^3^ and bacterial load reached values up to 6 × 10^3^ CFU/m^3^; in the indoor work site of the control workers group (automotive industry), the fungal load was < 3 × 10^2^ CFU/m^3^ and bacterial load was < 1.2 × 10^2^ CFU/m^3^. The bacterial load registered in the indoor tanneries environment was similar to those registered by Skóra et al [24] in indoor tanneries in Poland, which ranged between 1.2 × 10^3^ CFU/m^3^ and 3.7 × 10^3^ CFU/m^3^. Although the presence of microorganisms in the air is normal [25], the difference between microbial load from the tannery environment and automotive environment is significant, up to two orders of magnitude for fungi and one order of magnitude for bacteria; thus, the microbial load in the automotive environment could correspond to normal microbial load in the air. Microbiological loads in indoor tanneries exceed allowed limit values based on European requirements. For example, Swedish requirements, established concentrations of up to 500 CFU/m^3^ of bacteria and concentrations of up to 300 CFU/m^3^ of fungi as an acceptable level in enclosed environments [16]. The American Association of Industrial Hygiene published a residential and commercial standards guide for fungal spores concentration in internal environments, establishing a limit of 500 CFU/m^3^ and 250 CFU/m^3^, respectively [26]. Brazil has established regulations that the total quantity of microorganisms in air of indoor environments cannot exceed 750 CFU/m^3^ [17]. Singapore has established regulations that the bacteria concentration in indoor environments cannot exceed 500 CFU/m^3^ [27]. In any event, the allowed bacterial load in an indoor environment ranged between 5 × 10^2^ CFU/m^3^ and 7.5 × 10^2^ CFU/m^3^ and the allowed fungal load ranged between 2.5 × 10^2^ CFU/m^3^ and 7.5 × 10^2^ CFU/m^3^. Thus, bacterial and fungal load in the indoor automotive environment (work site of control group) complies with these European requirements, whereas bacterial and fungal load found in the indoor tanneries does not.

Skóra et al [24] concluded that the microbial contamination evaluation in the tanneries showed the increased bacteria and fungi numbers in the air in relation to outdoor air, which indicates an occupational inhalation risk to workers. The designated indicators of microbial contamination in the tanneries are associated with their working environment, specific and potentially pathogenic [24]. Thus, the high microbiological load in the working environment of the tanneries is evident.

Based on the poor air quality in the working environment of the tanneries and because many of these microorganisms can be acquired by the workers, either by inhalation, ingestion, or other means, some of the identified bacteria, in the indoor environment of tanneries, belonged to the following families: Bacillaceae, Corynebacteriaceae, Enterobacteriaceae, Moraxellaceae, Nocardiopsaceae, Pseudomonadaceae, and Staphylococcaceae. In addition, fungi genuses were also identified by microscopy and these included *Aspergillus* and *Penicillium,* which are considered the airborne allergenic fungi most significant and found to be associated with adverse effects on human and animal health [28]. Additionally, yeasts identified were *C. krusei* and *C. glabrata,* both of which have been associated with adverse health effects in individuals with compromised immune systems [13,29].

In this study, the characterization of the bacteria present in oropharyngeal mucosa revealed the presence of pathogenic bacteria in tannery employees, whereas the bacteria identified in the control group corresponded to normal flora. The most frequently identified family was Enterobacteriaceae, which was also present in the air at indoor tanneries [13].

Enterobacteriaceae pathogenicity has been reported in prior studies [30]. The identified species of this family included: *K. pneumoniae* which can reside in the respiratory tract and digestive system, and has been correlated with urinary tract infections, burns, diarrhea in neonates, and lung abscesses. *K. pneumoniae* is also considered an opportunistic pathogen that may cause bacteremia, pneumonia, and urinary tract infections. This is strongly correlated with nosocomial infections [31]. *E. aerogenes* and *E. cloacae* have also been correlated with urinary tract infections, as well as pneumonia and wound infections, while acting as a catalyst for other opportunistic infections. *E. aerogenes* is one of the most prevalent species found in clinical samples [32]. This pathogen is commonly found in wastewater, soil, and feces of animals and humans [33]. This pathogen has also been correlated with infections and bacteremia in hospitalized and nonhospitalized patients, and with the contamination of intravenous fluids in pediatric patients [34]. A prior study reported that this pathogen is known to have accounted for 25 of 58 episodes of bacteremia and 17 of 42 nosocomial bacteremia [32]. *E. cloacae* is also an opportunistic pathogen [31] that has been associated with opportunistic infections that affect the urinary and respiratory tract, as well as causing complications to skin wounds, in addition to the ability to cause septicemia and meningitis [35]. *Proteus* species are commonly associated with complicated urinary tract infections [36]. *P. vulgaris*, also considered an opportunistic pathogen, has been isolated from infected sites in immunosuppressed patients who have been receiving prolonged regimens of antibiotics [37]. *R. aquatilis* has been isolated mainly from water and clinical isolates are extremely rare, although it has been isolated from respiratory samples [38]. The infections ascribed to this organism included bacteremia, sepsis, respiratory infection, urinary tract infection, and wound infection in immunocompromised patients and infective endocarditis in patients with congenital heart disease [39]. *Citrobacter* species. have been found in water, soil, and the intestinal tract. These organisms cause a wide spectrum of infections in the respiratory tract and intestines [40]. In humans, this pathogen is associated with urinary tract infections, pneumonia, septicemia, and meningitis and other complications including respiratory diseases [41]. *Kluyvera* has been isolated from various clinical specimens, but its significance has not been clearly established. In fact, it has been regarded alternatively as saprophytic, opportunistic, or pathogenic [42]. *Kluyvera ascorbata* is described as an opportunistic pathogen [43]. *Enterobacter asburiae* has been found in human sources such as blood, urine, wounds, the respiratory tract, and feces [31]. This pathogen was reported in a case of interhospital pneumonia [44]. *E. coli* resides in the intestines of humans and animals, and has been observed in feces. The pathogenic strains have been known to cause urinary tract infections, sepsis, meningitis, and diarrhea [45]. Nonpathogenic strains have been correlated with opportunistic infections, such as pneumonia in hospitalized patients with immune suppressed metabolisms, as well as wound infection complications [37]. *S. marcescens* was reported as an opportunistic pathogen in one case of childhood meningitis, after the use of a disinfectant solution of benzalkonium chloride became contaminated. This pathogen isolated from human clinical is often associated with pneumonia and sepsis in patients with malignancies in the reticulum endothelial system and in turn have received chemotherapeutic agents [37].

Others studies have reported airborne fungi are of greater significance than bacteria as causes of allergic disorders such as rhinitis or asthma [28]. Our findings indicate that the Enterobacteriaceae family represents 57% of all identified bacteria in oropharyngeal mucosa of tanners. Enterobacteriaceae is a medically significant family, serving as a biological indicator of health risk.

Species belonging to Pseudomonadaceae, Neisseriaceae, Alcaligenaceae, Moraxellaceae, and Xanthomonadaceae families have been reported as opportunistic pathogens, such as *P. aeruginosa* [46–48], *N. bacilliformis* [49,50], *N. subflava* [51], *A. xylosoxidans* [52], *Alcaligenes faecalis* [53], *A. johnsonii* [54], and *Stenotrophomonas maltophilia* [55].

By contrast, identified bacteria within the control group are neither pathogenic nor opportunistic. *Neisseria polysaccharea* lives as commensal within humans. Studies found that *N. polyccharea* is located in the upper respiratory tract in approximately 0.5% of individuals, but not described as part of a pathological process [56]. *A. calcoaceticus* belongs to the family Moraxellaceae, which is a natural inhabitant of human skin and can also be commensal in the oropharyngeal mucosa [57].

In the tanners and control groups strains of *C. glabrata* and *C. albicans* were observed. However, in the tanners group, the yeasts *C. tropicalis* and *C. krusei,* were present, indicating an additional contaminate in the indoor tannery environment; these were not identified in the control group.

*Candida* species are the most common opportunistic fungal pathogens in humans, with *C. albicans* being the most prevalent pathogen correlated with mucosal and systemic fungal infections [58,59]. *C. albicans* is part of the normal microbial flora that colonizes mucocutaneous surfaces (oral cavity, gastrointestinal tract, and vagina) of the healthy human host. Although *Candida* does not normally cause disease, when immune defenses are compromised or the normal microflora balance is disrupted, *C. albicans* transforms itself into an opportunistic pathogenic killer [60], which has been associated in the pharynx with pneumonitis [61]. *C. glabrata* has been considered a nonpathogenic saprophyte found in the normal flora in healthy individuals; however, *C. glabrata* has emerged as an significant opportunistic pathogen present in the oral mucosa [62], and is increasingly prevalent in systemic infections in recent years [63,64].

*C. tropicalis* has been identified as the most prevalent pathogenic yeast species of the non-*C. albicans* group. Infections (candidiasis) caused by *C. tropicalis* have increased dramatically on a global scale, thus proclaiming this organism to be emerging as a pathogenic yeast [65].

*C. krusei* is an opportunistic pathogen that presents intrinsic resistance to fluconazole and has been described as a causative agent of disseminated fungal infections in susceptible patients [66].

In conclusion, although many of these microorganisms are considered within the normal flora in humans, many have become associated with the development of diseases. Some identified genera in oropharyngeal mucosa have already been reported in prior publications as airborne microbes in working environments, including *Klebsiella*, *Pseudomonas*, *Enterobacter*, *Citrobacter*, *Serratia*, and *Stenotrophomonas* [13,24,67].

The presence of pathogens and opportunistic pathogens in samples of oropharyngeal mucosa of tannery workers is indicative of workplace environmental pollution and poor hygiene practices, that over time can be detrimental to the health of the workers. In the tanners group, 42% of identified bacteria are correlated with respiratory affections; and the most abundant families (Enterobacteriaceae and Pseudomonadaceae) are correlated with diarrheal infections. These results are consistent with the findings cited in the epidemiological bulletin of the Secretaría de Salud del Estado de Guanajuato, Boletín Epidemiológico, Semana 30 22–27 Julio 2012, in which respiratory and diarrheal affections are the main causes of medical consultation [12]. By contrast, bacteria identified in the control group were not correlated with diarrheal and respiratory diseases, or other disorders.

With respect to the efficiency of the tested antibiotics, TZP had the higher efficiency and 90% of bacteria identified in the tanners groups were sensitive; followed by TIM, SAM, and AMC.

Based on the results of our study, we have determined that the microbiological contamination in the environment of the tanneries is evident and significant, and although some workers are asymptomatic, mitigation measures must be implemented to establish a healthy and safe working environment.

## Conflicts of interest

The authors declare that there is no conflict of interest.

## Figures and Tables

**Table 1:** Antibiotics and critical diameters

Antibiotic	Disc content (μg)	Diameter (mm)		
		S	I	R
Ticarcillin/clavulanic acid	75/10	≥ 20	15–19	≤ 14
Ampicillin/sulbactam	10/10	≥ 15	12–14	≤ 11
Amoxicillin/clavulanic acid	20/10	≥ 18	14–17	≤ 13
Piperacillin/tazobactam	100/10	≥ 21	18–20	≤ 17

I, immediate; R, resistant; S, sensitive.

**Table 2:** Antifungal and critical diameter interpretation for yeast sensitivity test

Antifungal agent	Disc content (μg)	Diameter (mm)	
		S	R
Fluconazole	25	≥ 19	≤ 14

R, resistant; S, sensitive.

**Table 3:** Workers (tanners and control) with a positive result to growth of bacteria and yeast

Group	Bacteria	Yeast
Tanners	63 (12/19)	63 (12/19)
Control	15 (3/20)	45 (9/20)

Data are presented as % (*n*/*N*).

**Table 4:** Identified bacteria from oropharyngeal mucosa samples of tanners group∗

Bacteria	Family
Tanners group	
*Kluyvera ascorbata*	Enterobacteriaceae
*Citrobacter murliniae*	Enterobacteriaceae
*Enterobacter asburiae*	Enterobacteriaceae
*Klebsiella pneumoniae*	Enterobacteriaceae
*Enterobacter*	Enterobacteriaceae
*Escherichia coli*	Enterobacteriaceae
*Rahnella aquatilis*	Enterobacteriaceae
*Serratia marcescens*	Enterobacteriaceae
*Proteus vulgaris*	Enterobacteriaceae
*Enterobacter aerogenes*	Enterobacteriaceae
*Pseudomonas brenneri*	Pseudomonadaceae
*Pseudomonas psychrotolerans*	Pseudomonadaceae
*Pseudomonas aeruginosa*	Pseudomonadaceae
*Neisseria bacilliformis*	Neisseriaceae
*Neisseria subflava*	Neisseriaceae
*Achromobacter xylosoxidans*	Alcaligenaceae
*Alcaligenes faecalis*	Alcaligenaceae
*Acinetobacter johnsonii*	Moraxellaceae
*Stenotrophomonas maltophilia*	Xanthomonadaceae
Control group	
*Neisseria polysaccharea*	Neisseriaceae
*Acinetobacter calcoaceticus*	Moraxellaceae

∗ Results of the control group are included.

**Table 5:** Identified yeasts from oropharyngeal mucosa samples of tanners group (identification through presumptive method, ChromAgar)∗

Yeast	Family
Tanners group	
*Candida glabrata*	*Candidaceae*
*Candida krusei*	*Candidaceae*
*Candida glabrata*	*Candidaceae*
*Candida glabrata*	*Candidaceae*
*Candida glabrata*	*Candidaceae*
*Candida tropicalis*	*Candidaceae*
*Candida glabrata*	*Candidaceae*
*Candida glabrata*	*Candidaceae*
*Candida albicans* Hb37†	*Candidaceae*
*Candida albicans* Hb37†	*Candidaceae*
*Candida albicans* YN50-151205†	*Candidaceae*
*Candida glabrata*	*Candidaceae*
*Candida albicans* L8278†	*Candidaceae*
Control group	
*Candida albicans* Hb20†	*Candidaceae*
*Candida glabrata*	*Candidaceae*
*Candida glabrata*	*Candidaceae*
*Candida albicans* L3805†	*Candidaceae*

∗ Results of the control group are included.

† Identified yeasts by sequencing of 5.8S ribosomal RNA gene.

**Table 6:** Variable response of bacteria to the tested antibiotics

Isolated	Antibiotics			
	TIM	SAM	AMC	TZP
Tanners group				
*Enterobacter asburiae*	S	S	R	S
*Enterobacter cloacae*	S	I	R	S
*Pseudomonas brenneri*	R	R	R	S
*Enterobacter aerogenes*	S	I	R	S
*Pseudomonas aeruginosa*	S	R	R	S
*Neisseria bacilliformis*	S	R	R	S
*Enterobacter asburiae*	S	S	R	S
*Klebsiella pneumoniae*	S	S	R	S
*Serratia marcescens*	S	I	R	S
*Enterobacter asburiae*	S	R	R	S
*Quality control: Escherichia coli ATCC 35218*	21	13	17	26

AMC, amoxicillin/clavulanic acid I, immediate; R, resistant; S, sensitive; SAM, ampicillin/sulbactam; TIM, ticarcillin/clavulanate; TZP, piperacillin/tazobactam.

**Table 7:** Response of yeast to fluconazole

Isolated	Fluconazole
Tanners group	
*Candida glabrata*	R
*Candida krusei*	S
*Candida glabrata*	S
*Candida glabrata*	S
*Candida glabrata*	S
*Candida tropicalis*	S
*Candida glabrata*	S
*Candida glabrata*	R
*Candida albicans* cepa Hb37	S
*Candida albicans* cepa Hb37	S
*Candida albicans* cepa YN50-151205	S
*Candida glabrata*	S
*Candida albicans* cepa L8278	R
Quality Control: *Candida albicans* ATCC 90028	32

R, resistant; S, sensitive.
